# Nitric Oxide as a Potential Adjuvant Therapeutic for Neuroblastoma: Effects of NO on Murine N2a Cells

**DOI:** 10.3390/vetsci7020051

**Published:** 2020-04-23

**Authors:** Jenna L. Gordon, Melissa M. Reynolds, Mark A. Brown

**Affiliations:** 1 Department of Chemistry, Colorado State University, Fort Collins, CO 80521, USA; jenna.short@colostate.edu; 2 Department of Chemistry, School of Biomedical Engineering, Colorado State University, Campus Delivery 1872, Fort Collins, CO 80523, USA; 3 Department of Clinical Sciences, Colorado State University, Fort Collins, CO 80523, USA; mark.brown@colostate.edu

**Keywords:** nitric oxide, neuroblastoma, cell viability, pediatric cancer

## Abstract

Neuroblastoma, the most common extracranial solid tumor in children, accounts for 15% of all pediatric cancer deaths. Pharmaceutical applications of S-Nitrosylation, which, under normal conditions is involved with a host of epigenetic and embryological development pathways, have exhibited great potential for use as adjuvant therapeutics in the clinical management of cancer. Herein, an evaluation of the impact of nitric oxide (NO) as a potent anticancer agent on murine neuroblastoma cells is presented. Excitingly cell viability, colony formation, and non-carcinogenic cell analysis illustrate the significance and practicality of NO as a cytotoxic anticancer therapeutic. Resazurin, WST-8 (2-(2-methoxy-4-nitrophenyl)-3-(4-nitrophenyl)-5-(2,4-disulfophenyl)-2H-tetrazolium, monosodium salt), and MTT (3-(4,5-Dimethylthiazol-2-yl)-2,5-diphyltetrazolium bromide) assays consistently displayed a moderate, ~20–25% reduction in cell viability after exposure to 1 mM *S*-Nitrosoglutathione (GSNO). A colony formation assay demonstrated that treated cells no longer exhibited colony formation capacity. Identically GSNO-treated Adult Human Dermal Fibroblasts (HDFa) exhibited no decrease in viability, indicating potential discrimination between neoplastic and normal cells. Collectively, our findings indicate a potential application for NO as an adjuvant therapeutic in the clinical management of neuroblastoma.

## 1. Introduction

Organismal development involves a host of regulatory mechanisms for directing highly conserved patterns in gene expression [1,2,3]. One such pathway involves epigenetic modifications, via posttranslational modifications, leading to synchronization in genetic patterns of expression [1,2,3]. These patterns of synchronization ultimately guide developmental pathways during embryogenesis. Although aberrations in posttranslational modifications are often associated with developmental diseases including tumorigenesis [4,5,6,7,8], posttranslational events can also be manipulated and/or targeted for positive clinical applications [9]. 

Although neuroblastoma, the most common extracranial solid tumor in children, accounts for only 8% of all pediatric cancer diagnoses, it is the cause of 15% of all pediatric cancer mortalities [10]. Tumors form in the sympathetic nervous system, largely originating in the adrenal glands [11]. Long-term disease prognosis is variable, dependent on a myriad of factors including but not limited to the stage of the disease, age of the patient, presence/absence of MYCN gene amplification, and chromosomal aberrations [12]. Treatment options for neuroblastoma reflect those of other malignant tumors and vary depending upon the stage of the disease as well as relevant risk factors. Due to the multitude and intricacy of disease progression and response to treatment, the International Neuroblastoma Staging System (INSS) was developed to classify the extent of the disease and impact the treatment approach [13]. 

Three distinct stages comprise the INSS: low, intermediate, and high risk. Each risk group is comprised of definitions containing the stage(s), patient age, presence/absence of the MYCN gene, histology, and DNA ploidy. Currently, low risk tumors represent ≤25% of initial diagnoses, intermediate risk represent about ~15% of initial diagnoses, and high risk tumors are the most commonly diagnosed, at ≥60% [11]. In patients with low risk disease, surgery and observation are the central therapeutic options that contribute to >90% survival [14,15]. Patients with intermediate risk disease typically receive a variety of sequential treatments including chemotherapy, surgery, and radiation therapy, if necessary. Survival rates in this group drop some but remain favorable at >80% [16,17]. High risk treatment protocols commence in an identical manner and additional treatments are frequently required, such as immunotherapy and bone marrow transplant. Despite the advances in treatments for low and intermediate risk disease, patients with high risk disease continue to face particularly poor prognosis, with ~40% five-year overall survival rates [18,19]. Overall relapse rates are additionally overwhelming, at >60% [12]. In a similar fashion, treatments for recurrent low-risk and intermediate-risk disease are largely effective. However, recurrent high-risk neuroblastoma remains a significant challenge. 

Due to the aggressive and unpredictable nature of high-risk and recurrent neuroblastoma, recent work has focused on the development of new therapeutic techniques [12]. The multitude of new therapies include cytotoxic agents, targeted agents, immunotherapy, retinoids, angiogenesis inhibitors, tyrosine kinase inhibitors, as well as other approaches [12]. One approach that has been of little focus in pediatric neuroblastoma research is the use of nitric oxide (NO) as an anticancer agent [20,21]. Numerous studies have shown that NO effectively induces tumor-specific cytotoxicity and pharmaceutical applications of *S*-Nitrosylation have exhibited great potential for use as adjuvant therapeutics in the clinical management of cancer [22,23,24,25,26,27,28,29,30,31]. The body’s physiological NO production is facilitated through three forms of the enzyme NO synthase (NOS), neuronal NOS (nNOS or NOS1), inducible NOS (iNOS or NOS2), and endothelial NOS (eNOS or NOS3). The routes that produce NO change under varying conditions (i.e., pH and temperature), require the use of different forms of NO synthase (NOS), and differ in production rates and expression patterns [29]. The variances in timing, longevity, and intensity of NO release by these different pathways ultimately determines the resultant biological consequences. Current research indicates that specific, localized concentrations of NO are required to regulate platelet aggregation while bursts of elevated concentrations of NO induce cytotoxicity [29]. Based on normal physiological processes, it is understood that surges of concentrated NO induce cytotoxicity. Furthermore, NO-releasing compounds such as *S*-Nitrosothiols, which are naturally occurring components in the body, provide prolonged, regulated release of NO. As such, *S*-Nitrosolglutathione (GSNO) was applied as the NO donor in this work.

Herein, the utility of NO as a potential adjuvant therapeutic for murine N2a neuroblastoma cells was identified. The effect of NO, delivered by GSNO, on N2a cells was assessed by exposing 1 mM GSNO to N2a cells for 24 h in the absence of light at 37 °C in a 5% CO_2_ incubator. Initially, therapeutic efficacy was assessed through three cell viability assays: resazurin, WST-8, and MTT. Each of these assays showed a modest 21–24% reduction in cell viability after therapeutic exposure. Colony formation assays further established the impact of NO on N2a cells, highlighting complete lack of colony formation capacity after the 24 h treatment period. Subsequently, a final cell viability assay was performed to determine the effect of 1 mM GSNO on non-carcinogenic HDFa cells. The results from this assay show that HDFa cells were not affected. Ultimately, the amount of NO available to N2a cells was explored by measuring the NO-release profile of 1 mM GSNO in PBS (0.54 ± 0.04 μmol NO in 24 h) and monitoring of the GSNO λ_max_ in 1 mM GSNO in complete DMEM at 336 nm (decreased GSNO peak over 24 h corresponding to decrease in available NO). Overall, NO impact on murine N2a cells is unexceptional as a stand-alone treatment. However, this level of impact coupled with the observed minimized impact on healthy cells suggests exciting potential of NO as an adjuvant, offering the prospect of reducing detrimental patient side effects.

## 2. Materials and Methods 

### 2.1. S-Nitrosoglutathione Synthesis

#### 2.1.1. Materials

Reduced glutathione (GSH; High purity) was purchased from VWR International (Radnor, PA, USA). Hydrochloric acid (HCl) and EPA vials were purchased from Thermo Fisher Scientific (Waltham, MA, USA). Sodium nitrite (99.999%, NaNO_2_) was obtained through Alfa Aesar (Ward Hill, MA, USA) and acetone (≥99.5%) was purchased through Sigma Aldrich (St. Louis, MO, USA). 

#### 2.1.2. Synthesis of S-Nitrosoglutathione (GSNO)

*S*-Nitrosoglutathione (GSNO) was synthesized through a previously developed synthesis. Briefly, GSNO was synthesized through the addition of NaNO_2_ to a solution of GSH in Millipore water and 2 M HCl. The GSNO mixture was reacted with constant stirring in an ice bath. After 40 min, the solution was treated with acetone and allowed to continue reacting with constant stirring in an ice bath. After 10 min, the resultant red solution was filtered, first with gravity filtration for 10 min and then vacuum filtration for 3.5 h to isolate the GSNO precipitate. The GSNO precipitate was washed successively with ice-water and acetone. The red filtrate solution was discarded and the filtered solid pink powder (GSNO) was kept and analyzed by UV-Vis spectrophotometry at 336 nm to ensure >95% purity. 

### 2.2. Cell Culture—N2a

#### 2.2.1. Materials

Murine neuroblastoma N2a cells were acquired from Dr. Seonil Kim at Colorado State University. Cells were maintained with Dulbecco’s Modified Eagle’s Medium (DMEM) w/L-glutamine purchased from Fisher Scientific (Hampton, NH, USA), supplemented with 10% EquaFETAL 100% U.S. Origin Bovine Serum obtained through Atlas Biologicals (Fort Collins, CO, USA) and 1% Penicillin-Streptomycin Solution purchased from Fisher Scientific (Hampton, NH, USA). 

#### 2.2.2. Method

Complete cell medias were prepared by adding 10% total volume fetal bovine serum and 1% total volume penicillin-streptomycin to DMEM media (complete DMEM). Initial stock cultures were prepared by quickly thawing 1 mL (10^6^ cells) in 37 °C water bath for 1–2 min. Once thawed, cells were added to 9 mL of pre-warmed, complete media in a 15 mL centrifuge tube. After centrifugation at 2000 RPM, 4 °C, 5 min, the supernatant was discarded, and the cell pellet was resuspended in 5 mL complete media and transferred to a sterile T-25 cm^2^ flask. The flasks were placed in a 37 °C, 5% CO_2_ incubator. Cells were appropriately provided 10 mL fresh medium every 24–72 h. Cells were additionally counted and split at appropriate intervals determined through both microscopic and macroscopic observation. 

### 2.3. Cell Culture—HDF 

#### 2.3.1. Materials 

Adult human dermal fibroblasts (HDFa; lot # 80616174) were maintained with fibroblast basal medium (FBM) supplemented with fibroblast growth kit—low serum and penicillin-streptomycin-amphotericin B, all purchased from American Type Culture Collection (Manassas, VA, USA). Trypsin/EDTA Solution for Primary Cells and Trypsin Neutralizing Solution were also purchased from American Type Culture Collection (Manassas, VA, USA).

#### 2.3.2. Method

Complete cell medias were prepared by adding the indicated volume of each supplement within the fibroblast growth kit–low serum. Initial stock cultures were prepared by quickly thawing 1 mL (7.1 × 10^5^ cells) in 37 °C water bath for 1–2 min. Once thawed, cells were added to 9 mL of pre-warmed, complete media in a T-25 cm^2^ flask. The flasks were placed in a 37 °C, 5% CO_2_ incubator. Cells were appropriately provided 10 mL fresh medium every 24–72 h. Cells were additionally counted and split at appropriate intervals determined through both microscopic and macroscopic observation.

### 2.4. In Vitro Cell Viability Assays

#### 2.4.1. Materials

Colorimetric Cell Viability Kit I – WST-8 (2-(2-methoxy-4-nitrophenyl)-3-(4-nitrophenyl)-5-(2,4-disulfophenyl)-2H-tetrazolium, monosodium salt) (WST-8) was purchased from PromoCell (Heidelberg, Germany). Trypan Blue Solution was purchased from Sigma-Aldrich (St. Louis, MO, USA). CellTiter-Blue Cell Viability Assay (Resazurin) and 3-(4,5-Dimethylthiazol-2-yl)-2,5-diphyltetrazolium bromide (MTT) were purchased from VWR International (Radnor, PA, USA).

#### 2.4.2. Method

Cells were plated in 100 μL increments (containing between 100,000-200,000 cells per mL (~1000–2000 cells/well) in 96-well plates. After 24 h, the media was aspirated and replaced with either 100 μL of media (Positive control—PC; ≥7 samples), 100 μL of 1 mM GSNO (Sample—S; ≥7 samples), or 100 μL of 1 mM GSH (Functional control—GSH; ≥7 samples). After an additional 24 h, the media was aspirated and replaced with 100 μL of fresh complete media before performing the appropriate cell viability assay. All absorbance measurements were collecting using a BioTek Synergy 2 Multi-Detection Microplate Reader. An average and standard deviation of the untreated PC cells was calculated and compared to the absorbance value of each measured sample. Analysis of variance (ANOVA) was performed to determine the statistical difference of the measured data. 

#### 2.4.3. Resazurin Assay

In the resazurin assay, N2a neuroblastoma and HDF cells were plated separately at 200,000 cells/mL in a 96-well plate. Following the steps stated above, 20 μL of warm resazurin was added to each well containing cells. The 96-well plate was incubated in a static incubator at 37 °C for 3.5 h (protocol indicates 1–4 h). Absorbance was measured at 570 nm and 600 nm using a microplate reader. In each experiment, data points were represented by an average (n ≥ 7) ± standard deviation.

#### 2.4.4. WST-8 Assay

In the WST-8 assay, N2a neuroblastoma cells were plated at 100,000 cells/mL in a 96-well plate. Following the steps stated above, 10 μL of warm WST-8 solution was added to each well containing cells. The 96-well plate was incubated in a static incubator at 37 °C for 3.5 h (protocol indicates 1–4 h). Absorbance was measured at 450 nm using a microplate reader. In this experiment, data points were represented by an average (n ≥ 9) ± standard deviation.

#### 2.4.5. MTT Assay

In the MTT assay, cells were plated at 100,000 cells/mL in a 96-well plate. Following the steps stated above, 10 μL of warm MTT solution was added to each well containing cells. The 96-well plate was incubated in a static incubator at 37 °C for 3.5 h (protocol indicates 1–4 h). After the incubation period, 75 μL of media was removed from each well, followed by the addition of 50 μL DMSO to each well. The plate was incubated at 37 °C for an additional 10 min. Absorbance was measured at 540 nm using a microplate reader. In each experiment, data points are represented by an average (n ≥ 7) ± standard deviation.

### 2.5. Colony Formation

#### Method

In the colony formation assays, cells were plated at 100,000 cells/mL in a 24-well plate in 1 mL increments. After 24 h, the media was aspirated and replaced with either 1 mL of media (Positive control—PC; ≥4 samples), 1 mL of 1 mM GSNO (Sample—S; ≥4 samples), or 1 mL of 1 mM GSH (Functional control—GSH; ≥4 samples). After an additional 24 h, the media was removed, and the cells were harvested via trypsin administration, centrifugation, and collection. At this point, cells were re-plated at 200 cells/ mL in a 24-well plate in 1 mL increments. Colony formation was qualitatively monitored daily and quantitatively measured weekly for three weeks by bright field microscopy. Colonies were defined as masses of at least 50 cells. In each experiment, data points are represented by an average ± standard deviation of n ≥ 3.

### 2.6. Nitric Oxide Analyzer Analysis of 1 mM GSNO in PBS

NO release over 24 h was measured at 1 min intervals in 1 mM GSNO in PBS. This measurement was performed in PBS instead of complete DMEM because the constant flow of N_2_ gas necessary for analysis caused foaming in experimental media that prevented the collection of NO release data. This was achieved using a chemiluminescence-based Nitric Oxide Analyzer (NOA 280i, GE Analytical, Boulder, CO, USA). The instrument was calibrated prior to operation by establishing a baseline with a nitrogen gas sweep followed by delivery of NO/nitrogen calibration gas at a flow rate of 200 mL/min. Immediately prior to data collection, 10 mM GSNO was prepared in a scintillation vial with protection from light and added directly into the NOA sample vessel containing PBS to achieve a final concentration of 1 mM GSNO. The bottom half of the sample vessel that contained solution was immediately submerged in a 37 °C water bath to promote NO release. Finally, the cell and water bath combination were covered with foil to eliminate photolytic decomposition. The NO release profile was ultimately used to determine the total NO release (in mol) of 1 mM GSNO in PBS in 24 h.

### 2.7. UV-Vis Analysis of 1 mM GSNO in Complete DMEM

NO release from 1 mM GSNO in complete DMEM was assessed using a Nicolet Evolution 300 UV-Vis spectrophotometer (Thermo Electron Corporation, Madison, WI, USA). A baseline was established prior to analysis using complete DMEM media. Absorbance data from 250 nm–600 nm was collected at 1 min intervals for 24 h to observe the absorbance at the λ_max_ of GSNO, 336 nm. A decrease in GSNO absorbance at 336 nm corresponds to the decrease in available NO.

### 2.8. Data Analysis/Statistics

All the data points are expressed as the average ± standard deviation. Statistical analysis was performed using one-way ANOVA. Statistically significant differences were defined at *p <* 0.01. 

## 3. Results

### 3.1. Cell Viability Assays

**Figure 1:**
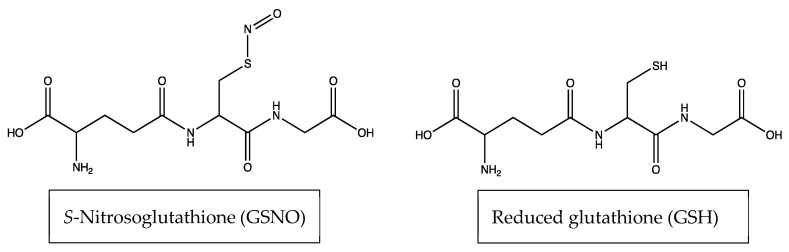
Structures of *S*-Nitrosoglutathione (GSNO) and reduced glutathione (GSH).

Cell viability assays were performed on murine N2a neuroblastoma cell lines to determine their response to 1 mM GSNO. Overall, resazurin, WST-8, MTT, and colony formation studies consistently showed decreased cell viability in N2a cells. Cell death was attributed to NO based-on control experiments run with reduced glutathione (GSH) in place of GSNO. Cells were also treated with GSH as a control experiment to highlight that GSNO—which is synthesized through the substitution of a NO group with the thiol group on GSH—is the only molecule (between the two aforementioned molecules) that impacts the viability of N2a cells (Figure 1). This indicates that NO, which is released spontaneously from GSNO (due to heat at 37 °C) is responsible for the observed decrease. It is not clear which reactions involving NO induce this response. Finally, cell viability of HDFa, analyzed with the resazurin assay, showed no decrease in viability after therapeutic application. These results exhibit the therapeutic potential of NO as an anticancer treatment against neuroblastoma.

#### 3.1.1. Resazurin Assay—Analysis of Cellular Viability

**Figure 2:**
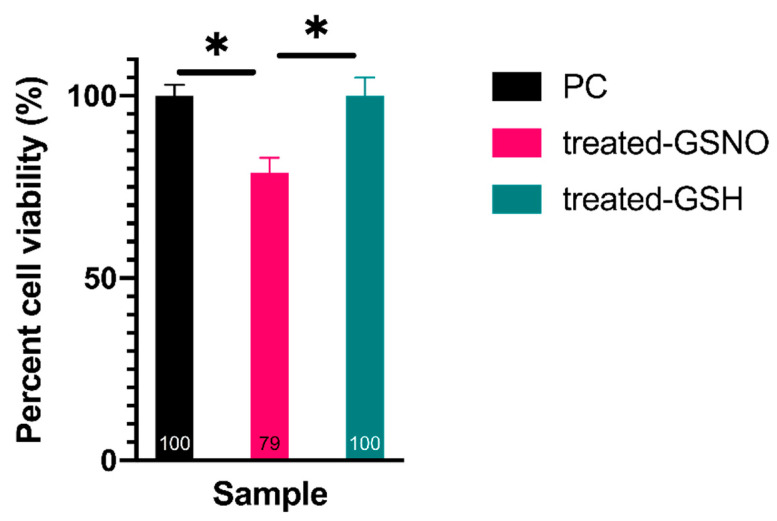
Percent cell viability of N2a neuroblastoma cells when exposed to 1 mM GSNO for 24 h at 37 °C, 5% CO_2_ assessed by the resazurin assay. Data points represent the mean of n ≥ 32 data points ± standard deviation. Treated-GSNO cells were determined to be statistically different than the positive control (PC) and the treated-GSH samples while the PC and treated-GSH samples were not statistically different at a 99% confidence interval (CI). Statistical significance at the 99% CI was illustrated graphically using * (1 asterisk).

The resazurin assay was implemented to assess the cellular viability of cells after GSNO-treatment. The results revealed a statistically significant, albeit moderate decrease in the viability, ~21%, of GSNO-exposed cells in comparison to untreated PC and treated-GSH cells (Figure 2). (Note: This data demonstrates an apparent impact on N2a neuroblastoma cells that can be directly correlated to NO release. Specifically, the effect of 1 mM GSNO on treated cells was determined through direct comparison to the untreated PC which was defined as 100% viable ± standard deviation (±3%). Using this method, cells treated with 1 mM GSNO were found to be 79% ± 4% viable. It is important to reiterate that treated GSH samples were found to be 100% ± 5% viable, indicating that NO was the active therapeutic, not GSNO. Studies done by Kim et al. as well as Suchyta and Schoenfisch on NO-based anticancer therapeutics have shown analogous results, indicating reductions in cellular viability in up to 60% of human SK-N-MC neuroblastoma cells and up to 66% in ovarian cancer cell lines, respectively [21,24]. Although moderate, the effect observed here is particularly promising as it demonstrates the potential of NO-releasing therapeutics to serve as adjuvants to other anticancer therapeutic approaches while decreasing detrimental side effects to the patient.

#### 3.1.2. WST-8 Assay—Analysis of Cellular Viability

**Figure 3:**
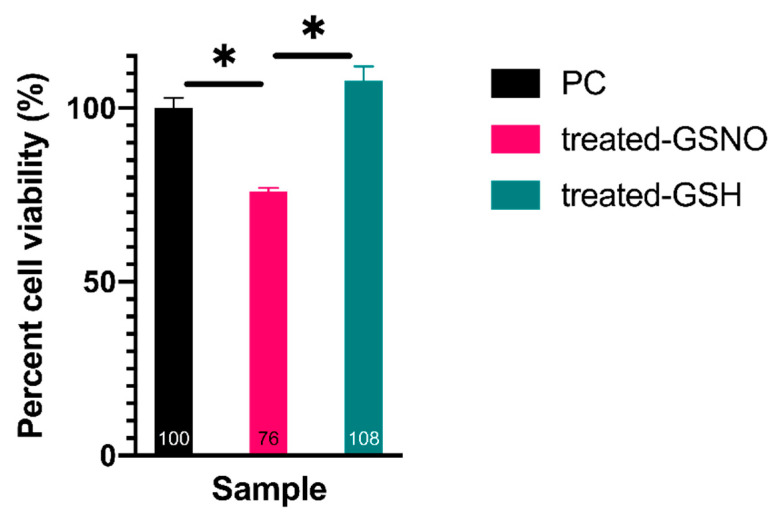
Percent cell viability of N2a neuroblastoma cells when exposed to 1 mM GSNO for 24 h at 37 °C, 5% CO_2_ assessed by the WST-8 assay. Data points represent the mean of n ≥ 9 data points ± standard deviation. One-way ANOVA results confirmed that treated-GSNO cells exhibited statistically different cellular viability than the PC and treated-GSH cells; the PC and treated-GSH samples were not statistically different at a 99% CI. Graphically, statistical significance at the 99% CI was illustrated using * (1 asterisk).

Another cell viability assay, the WST-8 assay, was performed to determine the impact of NO on N2a cells. The statistically different results demonstrated again that GSNO-exposed N2a cells decreased in cellular viability, ~24%, in comparison to untreated PC and treated GSH samples. In an identical fashion as with the resazurin assay, the non-treated PC sample was defined as 100% ± 3% viable. In comparison, cells treated with 1 mM GSNO were found to be 76% ± 1% viable, and treated GSH cells were found to be 108% ± 4% viable (Figure 3). This data further corroborates the previous claims: GSNO-treated cells moderately decrease in cellular viability and the observed decrease is due to NO-release from GSNO. Again, this result was in accordance with similar NO-based anticancer therapeutic studies [21,24] and demonstrates the potential of NO-based anticancer adjuvants.

#### 3.1.3. MTT Assay–Analysis of Cellular Viability 

**Figure 4:**
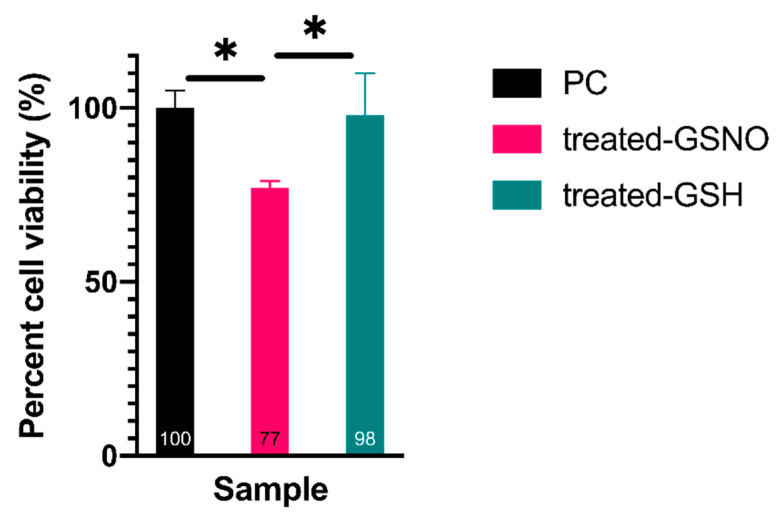
Percent cell viability of N2a neuroblastoma cells when exposed to 1 mM GSNO for 24 h at 37 °C, 5% CO_2_ assessed by the MTT assay. Data points represent the mean of n ≥ 7 data points ± standard deviation. One-way ANOVA revealed that treated-GSNO cells expressed a statistically different cellular viability than the PC and treated-GSH samples at a 99% CI. Conversely, PC and treated-GSH samples were not statistically different at a 99% CI. Again, statistical significance at the 99% CI was illustrated graphically using * (1 asterisk).

Finally, the MTT assay was used to confirm the impact of NO on cellular viability of N2a cells. These results mirrored the previous assays, highlighting a statistically significant decrease in cellular viability of GSNO-treated cells, ~23%, in comparison to untreated PC and treated GSH cells (Figure 4). Once again, the untreated PC sample was defined as 100% ± 5% viable. Excitingly, cells treated with 1 mM GSNO were found to be 77% ± 2% viable, and treated GSH cells were found to be 98% ± 12% viable. Ultimately, the observed cellular viability of N2a cells treated with GSNO assessed using each assay was remarkably similar, providing important evidence of the antitumor potential of NO in neuroblastoma treatment. The data observed here further corroborated the utility of NO-based anticancer therapeutics that has been presented in various other studies [21,24]. Clearly, the level of reduction identified in this study does not warrant a stand-alone anticancer treatment; however, NO has the potential to be exploited for use as an adjuvant.

### 3.2. Colony Formation Assay

**Figure 5:**
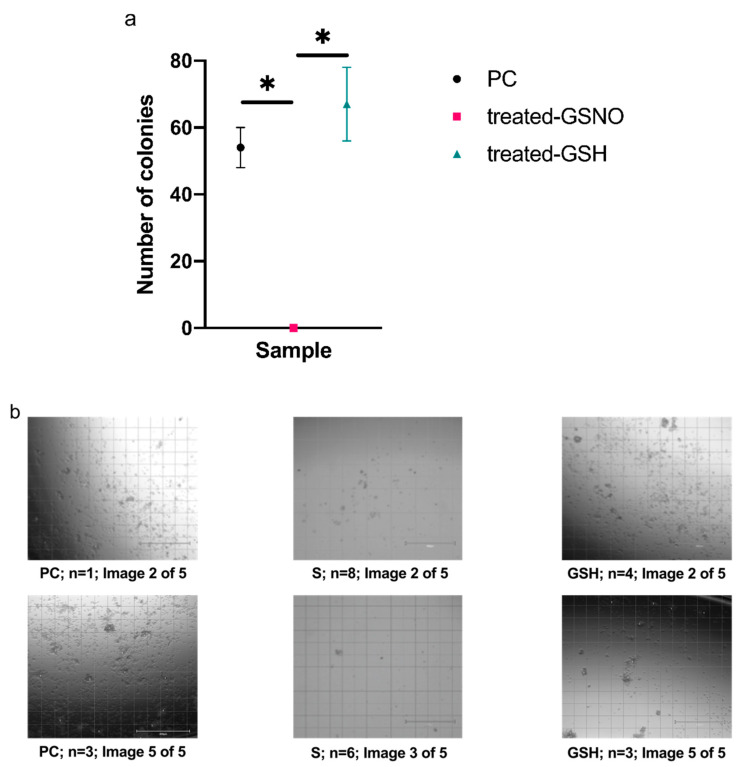
Colony formation capacity of N2a neuroblastoma cells after exposure to 1 mM GSNO for 24 h at 37 °C, 5% CO_2_. (**a**) Data points represent the mean of n ≥ 3 data points ± standard deviation. One-way ANOVA revealed a statistically significant difference between the treated-GSNO sample (0 colonies detected), the PC (54 ± 6), and treated-GSH (67 ± 11) samples while the PC and treated-GSH samples were not statistically different at a 99% CI. Graphically, statistical significance at the 99% CI was illustrated with * (1 asterisk). (**b**) Representative images of N2a cells after the colony formation period (3 weeks).

Colony formation assay results showed that untreated PC cells and GSH-treated functional control cells (n ≥ 3) retained colony formation capacity for 3 weeks following the 24 h treatment period. Explicitly, untreated PC cells formed 54 ± 6 colonies and GSH-treated cells formed 67 ± 11 (~16%). Although this number seems much higher than the PC, they were not statistically different based on one-way ANOVA at a 99% CI. Conversely GSNO-treated sample cells (n ≥ 8) formed 0 colonies in 3 weeks following the 24 h treatment period, which was statistically different than both the PC and the treated-GSH samples at a 99% CI. Despite some distinct variation in colony formation capacity of treated cells, it is most important to note that GSNO-treated N2a neuroblastoma cells consistently exhibited no colony formation capacity (Figure 5). [It is also important to reiterate that the colonies were defined as masses of at least 50 cells. No colonies larger than 50 cells were detected in the GSNO-treated cells.] Taken together, this information highlights the utility of NO-based anticancer therapeutics, exploiting both anti-proliferative and cytotoxic qualities.

### 3.3. Impact of NO on HDFa Cells—Resazurin Assay

**Figure 6:**
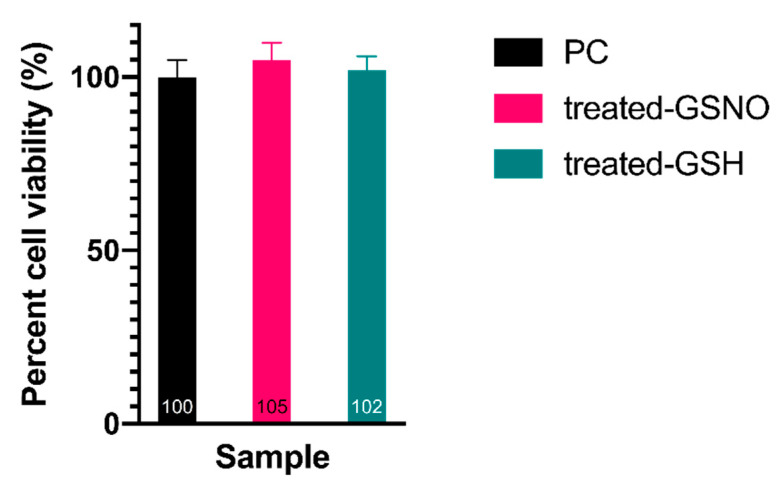
Percent cell viability of Human Dermal Fibroblasts (HDF) cells when exposed to 1 mM GSNO for 24 h at 37 °C, 5% CO_2_ assessed by the assessed by the CTB assay. Data points represent the mean of n ≥ 7 data points ± standard deviation. One-way ANOVA confirmed that all three sample sets did not statistically differ at a 99% CI.

Human dermal fibroblast cells served as a control to assess effect of NO on healthy cells. After 24 h exposure to 1 mM GSNO, the resazurin assay revealed no observable difference in cell viability of HDFa cells in comparison to nontreated HDFa cells (Figure 6). Cell viability of untreated PC was defined as 100% ± 5%. In comparison, cell viability of treated-GSNO was found to be 105% ± 5% and treated GSH was found to be 102% ± 4%. According to one-way ANOVA results, there was no statistical difference between any of the three samples tested at a 99% CI. These results provide valuable evidence that the impact of NO is higher on neoplastic cells than healthy cells. This phenomenon is extremely valuable as a potential means of decreasing detrimental side effects to patients, providing surplus justification of the utility of this therapeutic as an anticancer adjuvant.

### 3.4. NO-Release from 1 mM GSNO

#### 3.4.1. NO-Release from 1 mM GSNO in PBS

**Figure 7:**
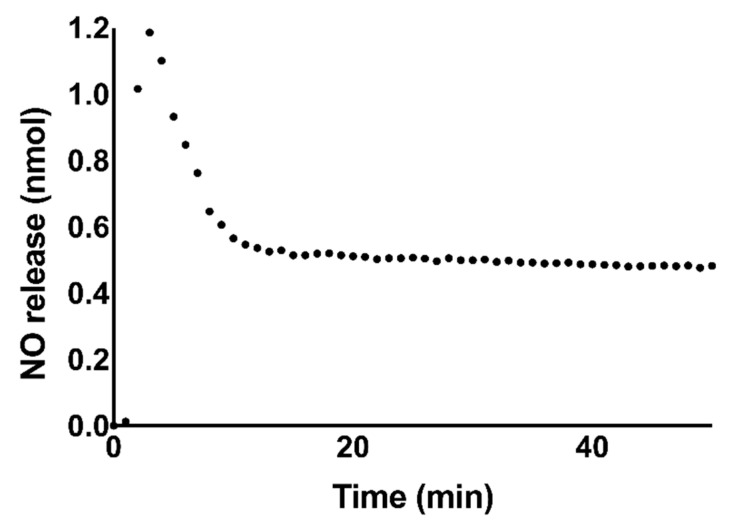
Representative graph of NO-release from 1 mM GSNO in PBS measured using a Nitric Oxide Analyzer (NOA). The total NO-release was measured over 24 h to be 0.54 ± 0.04 μmol NO. The first 50 min of NO-release data collection is shown here to highlight the initial burst of NO release in the first 10 min. After this period, the measured NO content remained nearly stable for the remainder of the 24 h measurement window. Data points represent an average of n = 3 data points.

The dosage and longevity of NO-release varies based on a variety of factors, such as donor composition, pH, presence of trace metal ions, light, and heat. These factors dictate the resultant biological consequences. In order to convey the amount of NO available to treated cells, NO-release was measured over 24 h in PBS using a Nitric Oxide Analyzer (NOA) (Figure 7). The total NO release in PBS was determined to be 0.54 ± 0.04 μmol NO over 24 h. This concentration of NO would be expected to induce apoptosis in neuroblastoma cells [29].

#### 3.4.2. Absorbance of GSNO λ_max_ via UV/VIS

**Scheme 1:**
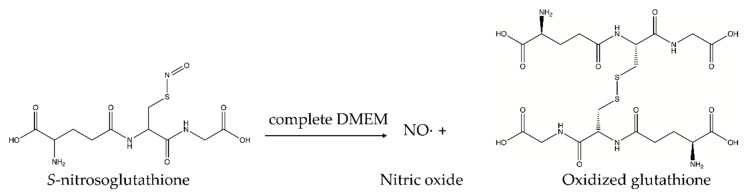
Hypothesized representation of the spontaneous decomposition of GSNO in complete DMEM to produce a nitric oxide radical (NO) and oxidized glutathione (GSSG).

To directly demonstrate the NO release profile in experimental media (complete DMEM) (Scheme 1), NO release was monitored via UV/Vis absorbance at 336 nm. In Table 1, it can be seen that the absorbance at the λ_max_ of GSNO, 336 nm, decreases over 24 h. This can be attributed directly to the decrease of GSNO over the 24 h period. It can also be seen that the absorbance at 336 nm decreases more rapidly than the chemiluminescent signal from 1 mM GSNO in PBS (Figure 7). This is likely due to a greater uptake and/or scavenging of NO in complete DMEM versus PBS. The data highlights that there is a finite quantity of NO available to cells and that it is mostly depleted in the initial 24 h exposure period.

## 4. Discussion

Nitric oxide (NO) is a small signaling molecule that is endogenously produced in the mammalian body. The various roles and interactions of NO in the body are extremely diverse and frequent, due to its small size and affinity for reactions. As such, its role in anticancer therapy is not straightforward and requires thorough investigation. Some of the important factors to address include the location, concentration, and release kinetics of NO [22]. Additionally, unlike traditional biosignaling molecules which typically influence cells through specific binding events, NO impacts cells via a multitude of potential reactions. The reactions involving NO that do occur depend on the aforementioned factors as well as the intra- and extracellular composition of cells in close proximity [20,21,22,23,24,25,26,27,28,29,30,31,32,33]. These reactions frequently result in the formation of reactive nitrogen species (RNS) such as peroxynitrite and nitrogen dioxide that can further influence cell health and longevity. Thus, effects of NO can be classified in two groups, direct (NO-mediated) and indirect (RNS-mediated) [34,35,36]. Overall, each of these factors impacts the effect of NO and RNS on neoplastic and healthy cells, varying extensively across cell types and strains.

Direct effects of NO can be attributed to rapid reactions between NO and metals, metal-oxygen species, and other radical species inside the cell membrane [34,35,36]. One pertinent reaction to consider is that of NO with the enzyme guanylate cyclase, which leads to the formation of an Fe(II)-nitrosyl complex. Once this complex is activated, it becomes cyclic guanosine monophosphate (cGMP), a mediator of various intercellular processes (i.e., platelet function, neurotransmission) [35,36]. Another important enzymatic reaction of NO results in the inhibition of cytochrome P450, which can lead to both positive (regulatory, protective) and negative (pathophysiological, repress drug metabolism) consequences [36]. Further, the reaction between NO and oxyhemoglobin is understood to play the primary role in regulation of biological movement and concentration of NO [34,36]. Finally, NO scavenging reactions with metal-oxygen species, lipids and other radical species result in tissue protective (NO + metal-oxygen complexes or lipids) and suppression of DNA synthesis and/or DNA-damage (NO + other radical species) [34,35,36]. Indirect effects of NO can be attributed to rapid reactions between NO and its derivatives with oxygen and superoxide to create RNS [34,36]. RNS are directly and indirectly impactful to DNA by creating additional carcinogens (nitrosamines), and influencing existing carcinogens [34,35,36]. NO further inhibits several DNA-repair enzymes [36]. Ultimately, the biological fate and influence of NO is highly variable. 

Due to the vast potential of direct and indirect NO-mediated impacts in physiological and pathological processes, it is not surprising that NO has been shown to induce tumoricidal and tumor-promoting effects [20,21,22,23,24,25,26,27,28,29,30,31,32,33,37,38,39]. Although seemingly contradictory, the evidence produced above provides rationale that these observations are logical. Specifically, current research indicates that one of the three forms of NOS, iNOS or NOS2, is most likely responsible for the tumor promoting and tumoricidal effects of NO [22,37,38,39,40]. NO generated via iNOS is involved in various cell processes, such as blood pressure regulation, inflammation, infection, and the onset and progression of malignant diseases [40]. The biological consequences range substantially depending on the occurrence and extent of iNOS expression. High levels of NO generated via iNOS generally exhibit apoptotic effects while low levels appear to be correlated to tumor progression and metastasis [22,37,38,39]. As such, this phenomenon can benefit researchers via manipulation of three factors according to the desired response: NO concentration, release kinetics, and location [22]. Although NO concentration can be directly manipulated, it is more challenging to control release kinetics and site-specific delivery of NO. To address these challenges various delivery platforms have been explored [23,24,25,26,27,28,29,30,31].

In anticancer applications the use of NO-based therapeutics is especially intriguing due to the accessibility and cost. Various NO-based therapeutics have been utilized in cancer research, including nitrated fatty acids, NO-loaded nanoparticles, and NO-donors [25,26,27,28,29,32,33,34,35,36,40]. NO-donors including diazeniumdiolates and RSNOs have been successfully applied to influence or control NO release kinetics in anticancer research [25,26,27,28,29,32,33,40]. Diazeniumdiolates, which consist of two moles of NO per molecule (vs one mole of NO per RSNO molecule), result in higher concentrations of NO-release than RSNOs. However, byproducts of the spontaneous decomposition are highly toxic and limit therapeutic potential. Particularly, RSNOs as NO-donor therapeutics are desirable since the mammalian body naturally produces and regulates many of these molecules. 

Based on this information, cell viability and colony formation assays were used in this research to emphasize the impact of the RSNO, GSNO on N2a neuroblastoma cells. Similar to previous studies [25,26,27,28,29,32,33], direct and indirect effects of NO were analyzed collectively (not distinguished) in this work. These experiments showed that after 24 h of exposure to GSNO, 21–24% of cells were no longer metabolically active and 100% were incapable of colony formation. The ability of high levels of NO to reduce colony formation has been previously observed [41]. Although the underlying molecular mechanisms for this effect are still being elucidated, it is known that excess NO impedes epidermal growth factor receptor/extracellular signal regulated kinase 2 (EGFR/ERK2)-dependent nuclear translocation of pyruvate kinase M2 (PKM2), thereby inhibiting glycolysis and inducing cell death [41,42]. It is also known that various NO-donor treated malignancies and macrophages have shown increased p53 expression, which led to apoptosis [35,36]. However, higher concentrations of NO have been found to decrease p53 expression, yet apoptosis can still be observed [36]. In this case, apoptosis may be influenced more by RNS-mediated DNA-damage and inhibition of DNA-repair [36]. 

Despite promising reduction rates in N2a cells and unaffected viability of HDFa cells, it was clear that this concentration of NO expresses the potential of NO as a discriminatory anticancer agent against N2a neuroblastoma cells. However, this current dosage cannot act as a stand-alone treatment against N2a neuroblastoma cells. Additionally, the modest reduction achieved in vitro would likely decrease in vivo. However, this data highlights the applicability and potential of NO as an anticancer agent against neuroblastoma cells and potentially other types of cancer. Practically speaking, there is the greatest potential that NO can be used as an adjuvant to chemotherapy or another anticancer therapeutic to increase efficacy and efficiency while decreasing detrimental effects to the patient.

Ultimately, supplementary investigation of NO as an anticancer therapeutic is warranted. Several potential avenues can be explored, including increased exposure time to 48 h and/or 72 h and increased therapeutic concentration. Additionally, the effect of NO can be assessed on various forms of neuroblastoma as well as other malignancies. The NO-based therapies can be combined with other cytotoxic or cytostatic therapeutics, such as pre-existing chemotherapeutics and/or alternative therapeutics, to assess synergistic and/or antagonistic effects. In this regard, it is also important to address the concept of NO generation in cancer cells potentially inducing chemoresistance in certain malignancies, (i.e., gliomas and pancreatic carcinomas) [37,38]. In the same manner as previously mentioned, development of chemoresistance due to endogenous production of NO has been linked to iNOS expression and production. It is possible that NO indirectly influences this phenomenon as its production upregulates release of an interleukin, such as (IL-1β), that instigates chemoresistance; possible solutions include inhibition of iNOS, which leads to significant reduction of chemoresistance, as well as targeted NO delivery [37,38,39]. Even with this information, it is still unclear exactly how NO impacts chemoresistance, tumor-promoting, and tumoricidal effects on various cancers. Additionally, it is unclear how the tumor composition and microenvironment influence these same factors. Thus, it is essential to explore the specific impact of NO (and the concentration) on various malignancies and phenotypes. 

In this study, there were only tumoricidal effects observed, no tumor-promoting effects detected, and chemoresistance and NO-mediated induction of apoptosis have not yet been explored. It is vitally important to assess these occurrences in N2a neuroblastoma as well as a range of neuroblastoma cell lines through apoptosis assays, RNA-sequence analysis, and eventually in vivo animal studies. Indeed, these studies show that NO, delivered by GSNO, is a promising adjuvant therapeutic in the treatment of neuroblastoma.

## 5. Conclusions

The majority of original neuroblastoma diagnoses confirm advanced stages, the majority of neuroblastoma patients will relapse after treatment, and the majority of relapsed patients will die from this disease. Obviously, it is essential to develop new methods to prevent, diagnose, and treat this deadly disease. Herein, analysis of NO as an anticancer agent was explored on murine N2a neuroblastoma cells. Ultimately, results from three different cell viability assays, resazurin, WST-8, and MTT, confirm the anticancer activity of NO when delivered through a donor-platform, GSNO. The results consistently revealed a decrease in N2a viability of 20–25% after exposure to 1 mM GSNO for 24 h. Colony formation assays additionally confirmed the lack of colony formation capacity after therapeutic exposure. Overall, NO, delivered via innate bodily compounds such as RSNOs, provides an exciting approach to anticancer therapeutics as it has the potential to increase efficacy of traditional therapeutic approaches while decreasing detrimental effects to patients. 

## Figures and Tables

**Table 1:** Absorbance at 336 nm (GSNO λ_max_) of 1 mM GSNO in complete DMEM over 24 h. This data exhibits the decline of GSNO absorbance, particularly in the first 18 h of analysis. Proportionally, the concentration of GSNO and available NO decline over the 24 h period.

Time (h)	Absorbance at 336 nm (GSNO λ_max_)	Concentration of GSNO (mM)	Amount of Available NO (μmol)
0 h	0.895	0.971	0.433
6 h	0.598	0.649	0.290
12 h	0.304	0.330	0.147
18 h	0.130	0.141	0.0630
24 h	0.135	0.146	0.0650
